# Structural Analysis of the 42 kDa Parvulin of *Trypanosoma brucei*

**DOI:** 10.3390/biom9030093

**Published:** 2019-03-07

**Authors:** Edisa Rehic, Dana Hoenig, Bianca E. Kamba, Anna Goehring, Eckhard Hofmann, Raphael Gasper, Anja Matena, Peter Bayer

**Affiliations:** 1 University Duisburg-Essen, Research Group Structural and Medicinal Biochemistry, Centre for Medical Biotechnology (ZMB), University of Duisburg-Essen, 45117 Essen, Germany; edisa.rehic@uni-due.de (E.R.); dana.hoenig@uni-due.de (D.H.); bianca.kamba@uni-due.de (B.E.K.); Anna.goehring@uni-due.de (A.G.); anja.matena@uni-due.de (A.M.); 2 Protein Crystallography, Faculty of Biology and Biotechnology, Ruhr University Bochum, 44801 Bochum, Germany; eckhard.hofmann@bph.rub.de; 3 Max Planck Institute for Molecular Physiology, 44227 Dortmund, Germany; raphael.gasper@mpi-dortmund.mpg.de

**Keywords:** *Trypanosoma brucei*, Parvulin, *cis*/*trans* Isomerase, PPIase, FHA

## Abstract

*Trypanosoma brucei* is a unicellular eukaryotic parasite, which causes the African sleeping sickness in humans. The recently discovered trypanosomal protein Parvulin 42 (*Tb*Par42) plays a key role in parasite cell proliferation. Homologues of this two-domain protein are exclusively found in protozoa species. *Tb*Par42 exhibits an N-terminal forkhead associated (FHA)-domain and a peptidyl-prolyl-*cis/trans*-isomerase (PPIase) domain, both connected by a linker. Using NMR and X-ray analysis as well as activity assays, we report on the structures of the single domains of *Tb*Par42, discuss their intra-molecular interplay, and give some initial hints as to potential cellular functions of the protein.

## 1. Introduction

Trypanosomes are unicellular eukaryotic parasites belonging to the class Kinetoplastea. Infection with trypanosomes causes various diseases in humans, including Chagas disease (*Trypanosoma cruzei*) and the sleeping sickness disease (*Trypanosoma brucei*). *Trypanosoma brucei* is transmitted to its human host by the bite of a tsetse fly carrying this parasite in its salivary gland. After transmission, the parasite can enter the cerebral fluid through the blood vessel walls, causing the life-threatening effects of sleeping sickness. In the blood stream, *T. brucei* cells proliferate and are taken up again by the bite of a tsetse fly, where the parasite cells (procyclic state) in the intestine transform their energy metabolism from anaerobic glycolytic to aerobic processes and migrate to the salivary gland. Once the parasite entered the central nervous system of its human host, therapy against the outbreak of sleeping sickness is challenging.

Recently, the nuclear protein *Tb*Par42 (molecular mass of 42 kDa) has been identified, of which RNAi-induced knockdown in procyclic cells of *T. brucei* inhibits proliferation of the parasite, suggesting a key role of the protein in cell growth [1,2]. Orthologous proteins are found in *T. cruzei* and *Leishmania* [2], as well as in *Chlamydomonas* [3]. Protein sequence alignment identified *Tb*Par42 as a two-domain protein, exhibiting an N-terminal flanked forkhead association (FHA) domain and a putative peptidyl-prolyl-cis/trans-isomerase (PPIase) domain [4] connected by a linker region. FHA domains are generally known as phosphopeptide recognition modules [5], although the *Tb*Par42 FHA domain has not yet been proven to have affinity to phosphorylated substrates. FHA domains play a critical role in cellular events such as DNA damage, DNA replication, or cell cycle progression [5,6,7]. Peptidyl-prolyl *cis*/*trans* isomerases are enzymes that accelerate the adjustment of the equilibrium of Xaa–Pro (Xaa being any amino acid) moieties within substrates. Sequence alignments showed that the *Tb*Par42 PPIase domain belongs to the family of parvulin PPIases [8], of which the human orthologues *h*Pin1 and *h*Par14/17 are structurally and functionally characterized [9]. In contrast, the three dimensional structure and the function of the FHA and PPIase domains of *Tb*Par42 are unknown. Although the PPIase domain shows a relatively high sequence alignment to *h*Pin1 (37.5% identity; 56.7% homology; see Supplementary Figure S3), Goh and coworkers have demonstrated that the PPIase domain lacks *cis*/*trans* isomerase activity against phosphorylated substrate peptides [1]. So far, no further functional roles of *Tb*Par42 have been suggested.

We used nuclear magnetic resonance spectroscopy (NMR) and X-ray analysis to evaluate the structures and dynamics of the isolated domains of *Tb*Par42 (1-383) and discuss their intra-molecular interplay. Moreover, we demonstrated the recognition of phosphorylated peptides by conserved motifs within the FHA domain, and tested the catalytic activity of the PPIase domain against a broad variety of substrates. Similarity searches using DALI [10] suggest structural homology of the FHA domain of *Tb*Par42 to FHA domains of nuclear RNA-binding proteins such as Dawdle/SNIP1, suggesting a cellular role of *Tb*Par42 in RNA binding/processing events.

## 2. Materials and Methods 

### 2.1. Expression of TbPar42 Constructs

The DNA sequence of *Tb*Par42 from *T. brucei* (SwissProt database no. Q57XM6) was purchased with *Escherichia coli* adapted codon usage in a PEX-K vector from Eurofins Genomics (Ebersberg, Germany). The constructs *Tb*Par42-full length (1–383), N-terminal extended FHA domain (NterFHA) (1–177), linker region (172–266), and PPIase domain (264–383) were PCR-amplified using DreamTaq DNA polymerase (Thermo Fisher Scientific, Darmstadt, Germany), the oligonucleotides listed in Table S5, and the synthetized *Tb*Par42 DNA as a template. The constructs were Apa1/Xho1 (Fast Digest from Fermentas, Germany) cloned into a modified pET-41 expression vector as described before [11], with an N-terminal GST-His_6_ fusion and a PreScission protease cleavage site. Before transformation into *E. coli* BL21(DE3)T1r (Sigma-Aldrich, Darmstadt, Germany) the constructs were verified by Sanger sequencing.

For production of unlabeled protein, 50 mL LB overnight culture (50 µg/mL kanamycin) was harvested, resuspended in 1 L LB medium, and grown up to OD_600_ = 0.8 at 37 °C, 160 rpm. For the expression of isotopically-labeled ^15^N-or ^13^C-^15^N protein, 500 mL LB medium with kanamycin was inoculated with 2% [*v*/*v*] of the overnight culture and grown to OD_600_ = 0.8. Subsequently, cells were pelleted, resuspended in 2 L M9 minimal medium supplemented with 1 g/L [^15^N] ammonium chloride and/or 4 g/L [^13^C] glucose, and grown to OD_600_ = 0.8. After induction of protein expression by addition of 0.2 mM IPTG, cells were incubated overnight at 30 °C (*Tb*Par42-fl), at 25 °C (NterFHA and linker), and at 37 °C (PPIase), followed by centrifugation (6000 rpm, 20 min, 4 °C). The triple labeled ^2^H-^13^C-^15^N-*Tb*Par42-fl protein was produced in M9 minimal medium where water was substituted with 99.8% D_2_O. The protein expression took 40 h at 30 °C after induction. Pelleted cells were resuspended in PBS buffer (NterFHA) or 50 mM BisTris, 150 mM NaCl pH 6.8 buffer supplemented with 1 mM PMSF for the other constructs, lysed with lysozyme (1 mg/mL) and disrupted by sonication. Upon ultracentrifugation (35,000 rpm, 60 min, 4 °C), the protein was purified by GSH affinity chromatography. PreScission protease digestion was used to cleave the GST fusion prior to size exclusion chromatography in 50 mM potassium phosphate, 150 mM potassium chloride, pH 6.3. For NMR studies, the protein was dialyzed against 50 mM potassium phosphate buffer pH 6.3, 90%/10% (*v*/*v*) H_2_O/D_2_O or 100% D2O, without additional salt.

### 2.2. Nuclear Magnetic Resonance (NMR) Spectroscopy

All spectra were recorded at 27 °C on a 700 MHz Ultrashield NMR spectrometer (Bruker, Rheinstetten, Germany) equipped with a triple (^1^H/^13^C/^15^N) cryoprobe. The NMR samples contained 200–600 µM of unlabeled or labeled protein, dissolved in potassium phosphate buffer. To the full length *Tb*Par42 sample, as well as to the linker construct, 2 mM dithiothreitol was added to the NMR sample. For the calibration of proton resonances 2,2-dimethyl-2-silapentane 5-sulfonate was used as an internal standard. ^15^N and ^13^C resonances were calibrated according to the IUPAC procedure [12,13]. Spectra needed for the assignment were recorded using standard Bruker library pulse sequences (except for the ^1^H-^15^N-HSQC-nuclear overhauser enhancement spectroscopy (NOESY)). Spectra were processed with the software Topspin 3.0 (Bruker) and analyzed with CcpNmr analysis [14]. For the assignment of the backbone and aliphatic side chain ^1^H, ^13^C, and ^15^N resonances, a set of 2D and 3D spectra (^1^H-^15^N-HSQC, ^1^H-^13^C-HSQC, ^1^H-^15^N- TOCSY-HSQC, HNCACB, CBCACONH, HNCA, HN(CO)CA, HNCO, HN(CA)CO) were recorded and analyzed. Aromatic side chain ^1^H and ^13^C chemical shifts were determined using two-dimensional ^1^H-^13^C-HSQC, ^1^H-^1^H-correlation spectroscopy (COSY), TOCSY and NOESY spectra (in H_2_O und D_2_O), and 3D ^1^H-^13^C-TOCSY-HSQC and ^1^H-^13^C-NOESY-HSQC spectra. Interproton distance constraints were obtained from the 2D ^1^H-homonuclear NOESY spectrum and the 3D ^1^H-^15^N-NOESY-HSQC. Hydrogen bond donors were derived from ^1^H-^15^N-HSQC spectra by proton/deuteron exchange experiments, after the protein was lyophilized and dissolved in 100% D2O. Acceptors were initially determined from homologue structures and, if applicable, were corrected during the calculation procedure. Hydrogen bonds were set as distance restraints of 1.7–2.6 for H-O and 2.7–3.5 for N-O. Torsion angle restraints were calculated with TALOS using sequence and chemical shift data as input [15]. Structure calculation from a random template structure was performed with the CYANA 2.1 software package including automated NOE assignment, simulated annealing, and torsion angle dynamic algorithm [16]. An ensemble of 60 structures was calculated, and the 10 structures with the lowest CYANA target function value [17] were selected. The secondary structure elements of the TbPar42-domains were defined on the basis of NOE connectivities [18], calculated distances, and H-bonding, as well as using chemical shift indices [19,20,21]. Peptides for NMR studies were purchased from CASLO (Lyngby, Denmark).

### 2.3. NMR Titration Experiments

To 440 μM ^15^N-labeled NterFHA or PPIase in 50 mM potassium phosphate buffer, pH 6.26, 90%/10% (*v*/*v*) H_2_O / D_2_O, the peptide Suc-Ala-pThr-Pro-Ala-NH_2_, dissolved in the same buffer, was added stepwise to a final concentration of 13 mM. After each step a ^1^H-^15^N-HSQC or SOFAST spectrum was recorded on a 700 MHz Ultrashield NMR spectrometer at 27 °C. The spectra were analyzed with the CcpNmr Analysis software, and the chemical shift perturbations were calculated with following equation [22]:

$$\Delta \delta_{total}=\sqrt{{\left(\Delta \delta_{H}\right)}^{2}+\left(0.154\cdot \Delta \delta_{N}\right)²}$$

Residues with chemical shifts ≥0.015 ppm (PPIase) or ≥0.04 ppm (NterFHA) were represented on the related protein structure using PyMOL [23]. For titrations of the peptide Ala-Glu-Ala-pThr-Glu-Ala-Xaa (Xaa representing Asp, Ile, Val, Ser, or Gln) to the NterFHA domain, the protein concentration was 250 µM and the peptides were titrated to a final concentration of 8 mM. 

### 2.4. EXSY Experiment

EXSY spectra were recorded for a high concentrated peptide probe (Suc-Ala-pThr-Pro-Ala-NH2 of 2.4 mM in 25 mM potassium phosphate buffer, pH 6.26, 100% D_2_O at 27 °C on a 700 MHz Ultrashield NMR spectrometer, in the presence and absence of 50 μM *h*Pin-PPIase or *Tb*Par42-PPIase. The pulse sequence used for the spectra recording was from the standard Bruker library, noesygpph19 with a mixing time of 400 ms, a relaxation delay of 1 s, and 16 scans.

### 2.5. Relaxation Measurements

The longitudinal (R_1_) and transverse relaxation (R_2_) rates were estimated by signal intensity measurements from a series of ^1^H-^15^N-HSQC spectra with varying relaxation delays. Ten different delay times were used to determine the R1 rate: 20, 100, 140, 180, 200, 300, 500, 750, 1000, and 1400 ms, whereas nine evolution times 10, 30, 50, 70, 90, 110, 130, 170, and 190 were used to estimate R_2_. R1 and R2 correspond to the reciprocal relaxation times T1 and T2, which were calculated from intensity measurements for each residue according to Reference [24]. The ^1^H-^15^N-NOE for each amino acid was determined from the ratio of signal intensities of a saturated and an unsaturated spectrum. The two spectra were extracted out of a simultaneously recorded spectrum, recorded with a modified hsqcnoef3gpsi pulse sequence. All measurements were recorded at 27 °C on a 700 MHz Ultrashield NMR spectrometer.

### 2.6. Sample Preparation for TbPar42-PPIase Crystallization Experiments

For crystallization, *Tb*Par42-PPIase (264–383) was purified by size exclusion chromatography using a Superdex_TM_ 75 16/60 pg (GE Healthcare, Freiburg, Germany) column equilibrated with 50 mM Tris/HCl, pH 7.0, 150 mM NaCl, 1 mM DTT. The fractions containing pure *Tb*Par42-PPIase were pooled and concentrated to 15–20 mg/mL. Crystallization conditions were screened by the hanging drop vapor diffusion method, mixing 2 µL reservoir solution with 2 µL protein solution incubated at 20 °C. Crystals used for structure determination grew at 20 °C in drop, with 23% PEG 4000, 100 mM sodium acetate as the reservoir. Prior to freezing in liquid nitrogen, crystals were soaked in 50% PEG 4000 for cryoprotection.

### 2.7. Data Collection and Structure Determination

Data sets were collected at 100 K on beamline P13 at PETRAIII of the Deutsches Elektronen Synchrotron (Hamburg, Germany) using a Pilatus 6M detector. The data were processed using the XDS suite and converted into appropriate formats by XDSCONV [25,26]. The *Tb*PPIase structure was solved by molecular replacement, using human Pin1 as a search model (PDB: 1Pin, residues 50–163) in MOLREP [27]. Refinement was done with REFMAC [28] and PHENIX [29]. For manual model building, the molecular graphics program COOT was employed [30]. The final model was deposited in the PDB with the accession code 6GMP.

### 2.8. PPIase Activity

A protease coupled isomerase assay [31,32] was used to measure the activity of the PPIase for peptides with a Suc-Ala-Xaa-Pro-Phe-pNA scaffold (pNA-paranitroaniline; Xaa-represents pSer, pThr, Arg, Lys, Glu, Asp, Phe, Gln, or Ser), synthetized by CASLO or ChinaPeptides. The peptides were dissolved in 0.5 M LiCl in 2,2,2-trifluorethanol to a concentration of 15 mM, followed by overnight incubation. The protease α-chymotrypsin (35 µM) was pre-incubated with 200 nM or 10 μM *Tb*Par42-PPIase (as positive control 20 nM *h*Pin1-PPIase was used) for 5 min in PBS buffer, pH 6.8 at 10 °C. For the mutants, 2 µM and 10 µM protein were used. Directly after the addition of 75 μM peptide, the assay was followed by monitoring the absorbance of the cleaved pNA at 390 nm. The reaction rate constant of the protease and the cis-isomer content of the peptide were determined from the uncatalyzed reaction for each substrate. The observed curves were analyzed with GraphPadPrism 5.04, using a bi-exponential reaction fit. 

## 3. Results

### 3.1. NMR Measurements of Full-Length TbPar42 (1–383)

**Figure 1:**
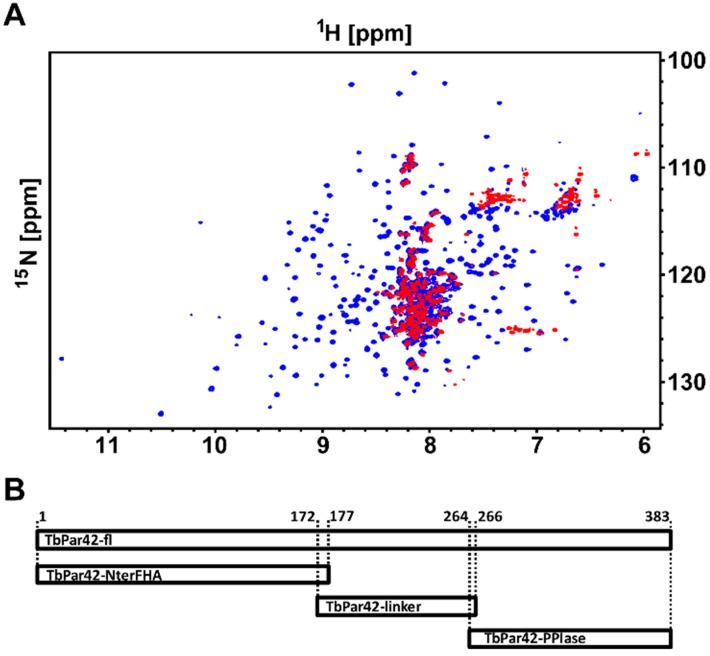
Nuclear magnetic resonance (NMR) spectroscopy and construct design of *Tb*Par42. (**A**) Superposition of a ^1^H-^15^N-total correlation spectroscopy (TROSY) of ^2^H-^13^C-^15^N-*Tb*Par42 (blue) and a ^1^H-^15^N-hetero single quantum coherence (HSQC) of ^15^N-*Tb*Par42 (red). (**B**) Design of expression constructs (horizontal bars) for full length protein (*Tb*Par42-fl), N-terminus with forkhead association (FHA) domain (*Tb*Par42-NterFHA) and PPIase domain (*Tb*Par42-PPIase). The corresponding residue numbers are given at the top of Figure 1B.

Due to the high molecular weight of *Tb*Par42 (1–383), structural investigations of the protein by NMR spectroscopy were only possible using ^2^H-^13^C-^15^N isotopically labeled protein harvested from *E.coli* grown in deuterium oxide. Although nearly 96% of all expected amide resonances were present in the ^1^H-^15^N-total correlation spectroscopy (TROSY) spectra (Figure 1A), the low protein yield upon recombinant production hampered a complete structural analysis. We therefore decided to dissect the full-length protein in separated domains, which were more easily amenable for NMR spectroscopy. These domains were primarily defined using sequence alignment and similarity searches (BLAST). After several rounds of trials and optimization (expression values, NMR spectra appearance), three feasible domain constructs of *Tb*Par42 (the N-terminal extended FHA domain (1–177; NterFHA), the putative linker region (172–266; Loop_FHA_PPIase), and the putative catalytic domain (264-383; *Tb*Par42-PPIase) were separately cloned, produced, and further analyzed (Figure 1B).

### 3.2. NMR Structure Calculation of TbPar42-PPIase

**Figure 2:**
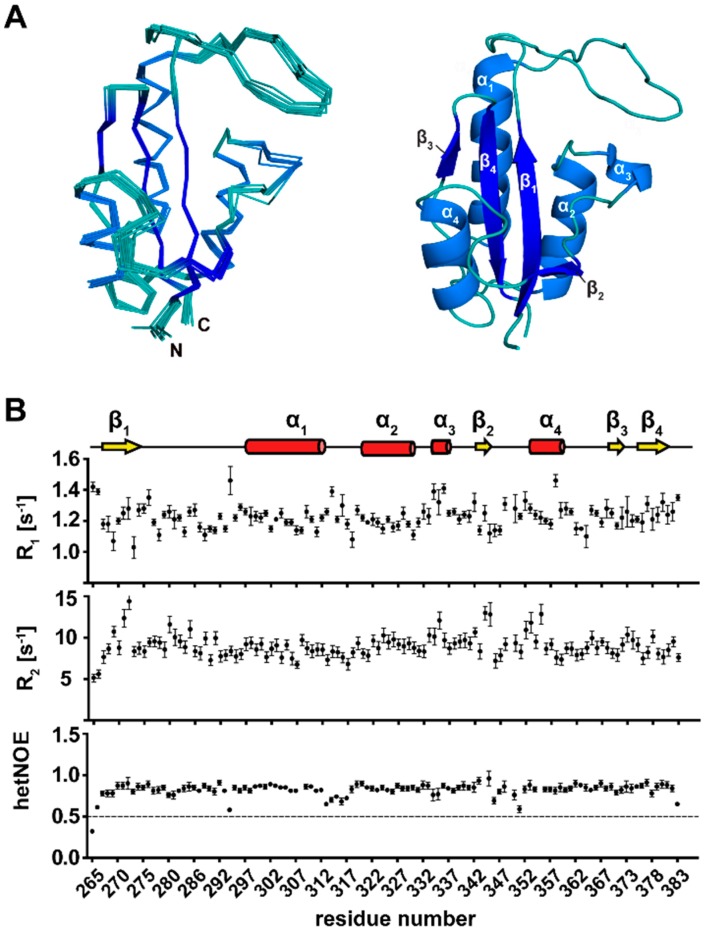
Structural and dynamical data of the PPIase domain of *Tb*Par42. (**A**) Ensemble of the 10 lowest energy structures (left) and ribbon representation of *Tb*Par42-PPIase (right). The four α-helices and four β-sheets of the parvulin fold are indicated. (**B**) Dynamics of the PPIase domain. The secondary structure elements are indicated at the top of the plot. Below, the longitudinal (R1) and transversal (R2) relaxation rates, as well as the HetNOE of the amino group of residues, within the domain are plotted.

60 structures of the *Tb*Par42-PPIase domain were calculated using 3023 distance NOEs, 206 torsion angle restraints, and 46 hydrogen bonds. A statistical analysis of the NMR ensemble (PDB-ID: 2N87; BMRB-ID: 19904) is shown in Table S1. Ten structures with the lowest energy conformations are presented in Figure 2A. 

### 3.3. Secondary Structure Elements of TbPar42-PPIase

The PPIase is buildup of four α-helices and four β-strands (Figure 2A,B). The first N-terminal β-strand comprises amino acids from Arg267 to Val274, and is followed by a long loop, which extends from Lys275 to Arg296. The adjacent α-helix 1 (Ser297 to His312) is separated through a small stretch of residues from α-helix 2 (Leu320–Phe330). Within the following thirteen residues, α-helix 3, which comprises only four residues (Gly334–Lys337), was identified by chemical shift data. Due to a lack of a typical helix-NOE pattern and fast exchanging amide protons in this helix, it is likely to be considered as α-helical turn. The second β-strand from Gly343 to Val345 is connected to α-helix 4 (Gly353 to Phe359) by a short loop. β-strand 3 is formed from Val370 to Thr372, and is followed by a short loop that turns into the fourth β-strand (Gly375–Glu381). Analysis of ^15^N-relaxation data reflecting the motion of the backbone N-H vector (Figure 2B) indicated that all secondary structure elements are rigid, and even the extended PPIase loop regions (β1-α1 and β2-α4) lack significant internal mobility.

### 3.4. The Tertiary Structure of TbPar42-PPIase Comprises the Typical Parvulin Fold

The PPIase adopts a globular fold, which is characteristic for parvulin type proteins [33]. The central β-sheet, composed of four antiparallel β-strands (β3-β4-β1-β2), is surrounded by four α-helices (Figure 2A). The dipole axes of these helices align parallel with the C–N orientation vector of the β-sheet. According to the DALI program, the solution structure of the PPIase domain of human parvulin *h*Pin1 (PDB-ID: 1NMW), an isomerase acting on phosphorylated substrates, has the highest similarity to the *Tb*Par42-PPIase (Z-score of 13.9; Cα r.m.s.d: 1.58 Å). 

**Figure 3:**
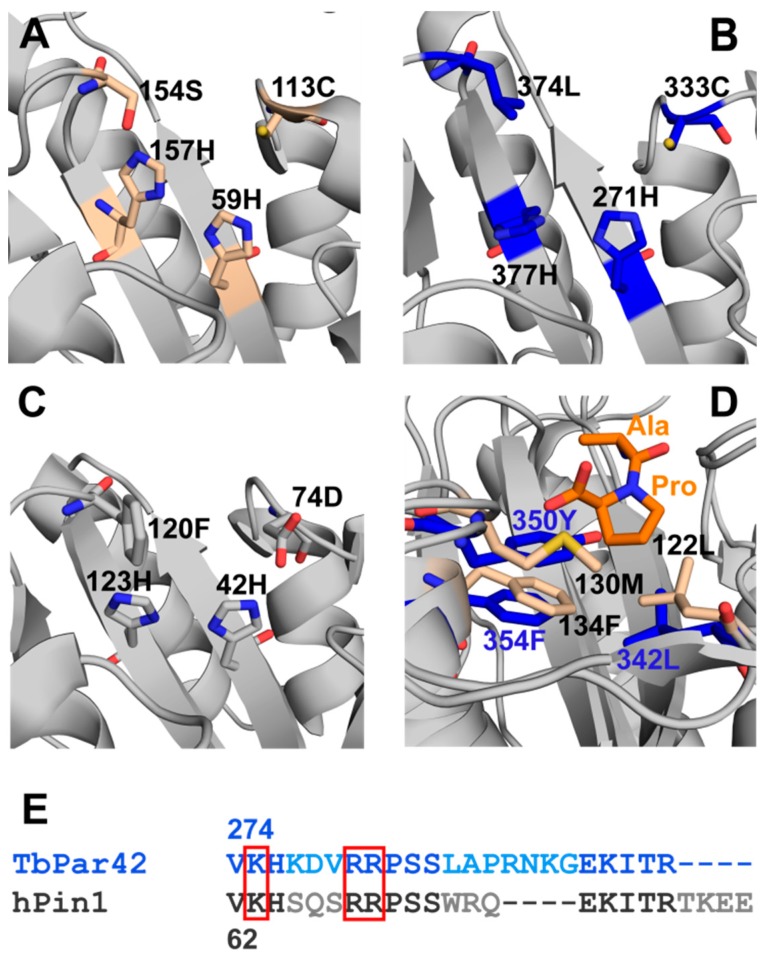
Structural insights into the PPIase domain of *Tb*Par42. Comparison of the catalytic tetrad (prominent residues are represented as sticks) of (**A**) *h*Pin1, PDB:1NMW, (**B**) *Tb*Par42, PDB:2N87, and (**C**) *h*Par14, PDB:3UI4. (**D**) Overlay of the solution structure of *Tb*Par42-PPIase (PDB:2N87) and the crystal structure of *h*Pin1 (PDB:1PIN), with its bound Ala-Pro dipeptide in orange. Hydrophobic residues of the proline-binding pocket are represented as sticks in blue for *Tb*Par42, and in beige for *h*Pin1. (**E**) Sequence alignment of the β1-α1 loop between *Tb*Par42 (blue) and *h*Pin1 (black). The phosphate group binding residues of *h*Pin1 are framed (red). A full alignment of both PPIase domains is found in Figure S3.

A comparison of both structures (Figure 3A,B) indicates that well-conserved residues of Pin1-like parvulins, e.g., the histidine motif (His59 and His157 in *h*Pin1; His271 and His377 in *Tb*Par42), as well as a characteristic cysteine within the catalytic cleft (Cys113 in hPin1; Cys333 in *Tb*Par42), are located at the same spatial positions within both folds. The *h*Pin1 Ser154 (Figure 3A), described as a part of the catalytic tetrad [34] is replaced by Leu374 in *Tb*Par42 (Figure 3B). In the catalytically active but non-phosphorylation specific parvulin *h*Par14, a phenylalanine (Phe120) can be found at this position (Figure 3C). Thr372, the counterpart of which in Par14 was associated with the catalytic network [35], is also conserved in the *Tb*Par42-PPIase structure. Residues Leu122, Met130, and Phe134 of Pin1, which are located at the concave side of the β-sheet core, constitute a hydrophobic binding pocket for the substrate’s proline ring moiety. At the same spatial positions, Leu342 and Phe354 can be found in the *Tb*Par42-PPIase structure, whereas the methionine is exchanged by a tyrosine (Tyr350), Figure 3D. Phosphorylation-specific parvulins exhibit an extended α1-β1 loop, with a basic amino acid cluster important for the binding of the phosphate group of substrates. A homologous loop is also present in the structure of *Tb*Par42-PPIase (Figure 3E). 

### 3.5. TbPar42-PPIase Lacks Catalytic PPIase Activity

Although the PPIase domain of *Tb*Par42 adopts a parvulin fold and reflects structural elements of Pin1-type proteins [1], it is catalytically inactive. 

**Figure 4:**
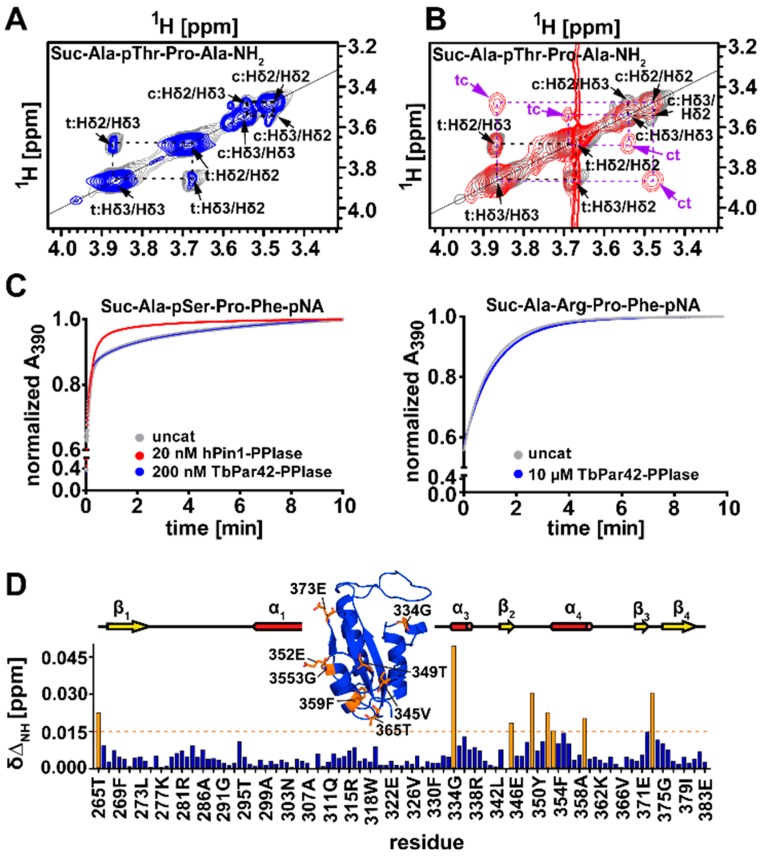
Catalytic activity and substrate binding of the PPIase domain. Exchange spectroscopy (EXSY) spectra of the proline-Hδ protons of Suc-Ala-pThr-Pro-Ala-NH_2_ peptide. (**A**) in the presence of *Tb*Par42-PPIase; (**B**) in the presence of *h*Pin1. The *cis* and *trans* conformers are indicated by c and t, and the resulting exchange peaks generated exclusively in B during isomerization are represented in purple by ct and tc. (**C**) Absorption curves monitoring the time dependent cleavage of pNA-containing model peptides (left: Suc-Ala-pSer-Pro-Phe-pNA (*h*Pin1) and right: Suc-Ala-Arg-Pro-Phe-pNA (*Tb*Par42-PPIase)) in a protease-coupled isomerase assay in the presence of *Tb*Par42-PPIase (blue) or hPin1 (red). The gray curve belongs to the uncatalyzed reaction without enzyme. A steep initial slope describes a fast isomerization. (**D**) Diagram of NH chemical shift changes (∆δ_NH_) observed in an ^15^N-HSQC spectrum of *Tb*Par42-PPIase versus amino acid sequence after addition of 13 mM Suc-Ala-pThr-Pro-Ala-NH_2_ peptide. Residues with a chemical shift larger than 0.015 ppm (orange dash line) are colored orange and mapped to the structure of the *Tb*Par42-PPIase domain. The secondary structure of *Tb*Par42-PPIase with helix represented in red, β-strand in yellow, and coil as line is shown on top of the diagram.

The protein failed to compensate the loss of ESS1 function in yeast (as *h*Pin1 does), and also lacked isomerase activity against a pThr containing substrate, which was used to verify the activities of *Tb*Pin1 and *At*Pin1 [1]. We confirmed this lack of activity of *Tb*Par42-PPIase by exchange spectroscopy (EXSY) experiments using an alternative substrate (Figure 4A,B), and by isomerase activity studies using a protease coupled assay (Figure 4C). In the latter experiment, a considerable ensemble of substrates containing pSer or pThr residues preceding proline were used to track activity. In addition, peptides containing negatively charged (Glu, Asp) positively charged (Arg), or uncharged (Ser, Gln, Phe) residues at the Xaa-Pro motif were assayed to test a putative sequence dependency of *Tb*Par42’s substrate activity for non-phosphorylated motifs (Figure 4C(right)). However, no *cis*/*trans*-activity could be detected against any of these substrates. From all the current data, we may conclude that the PPIase domain of *Tb*Par42 seems to have no enzymatic function within the protein. Although *Tb*Par42 seems to be catalytically inactive, titration experiments with a Suc-Ala-pThr-Pro-Ala-NH_2_ peptide showed chemical shift perturbations in the 2D ^1^H-^15^N hetero-single-quantum-coherence (HSQC) spectra of the PPIase domain, Figure 4D. Thus, the substrate was capable of binding to the enzyme. Residues affected by peptide addition were in near proximity to the proposed proline binding site (Thr265, Gly334, Val345, Thr349, Glu352, Gly353, Phe359 Glu373), but K_D_ values were in the millimolar range (~10 mM). 

### 3.6. What Is the Structural Origin of the Lack of Catalytic Activity of TbPar42-PPIase?

The structural fold and the spatial organization of amino acids in the putative catalytic center of *Tb*Par42-PPIase resemble the typical active side of a Pin1-type parvulin isomerase. However, despite the fact that binding of the model peptide Suc-Ala-pThr-Pro-Ala-NH_2_ seems to occur at the catalytic cleft close to the proline binding pocket (Figure 4D), no isomerase activity could be detected under the tested conditions (Figure 4A,C). Certainly, the dissociation constant of the peptide was only in the millimolar range, but this is no sufficient argument to explain the lack of activity, as comparable weak binding affinities have been pointed out for active parvulins and their substrates [36,37]. Thus, insufficient binding of substrates may not be the origin of catalytic inactivity. 

**Figure 5:**
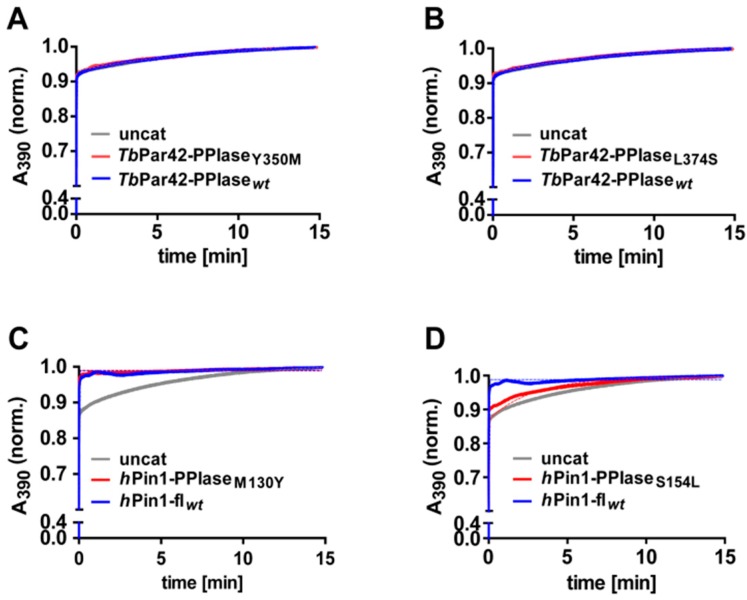
PPIase assays using 75 µM of the peptide Suc-Ala-pThr-Pro-Phe-pNA in the presence and absence of different PPIase constructs. Absorption curves of uncatalyzed reactions are highlighted in gray color. Catalyzed reactions are shown (**A**) in presence of 10 µM *Tb*Par42-PPIase_Y350M_ (red) and 10 µM *Tb*Par42-PPIase_wt_ (blue); (**B**) in presence of 10 µM *Tb*Par42-PPIase_L374S_ (red) and 10 µM *Tb*Par42-PPIase_wt_ (blue); (**C**) in presence of 10 µM *h*Pin1-PPIase_M130Y_ (red) and 2 µM full length (fl) *h*Pin1-fl_wt_ (blue); (**D**) in presence of 10 µM *h*Pin1-PPIase_S154L_ (red) and 2 µM full length (fl) *h*Pin1-fl_wt_ (blue).

More reasonably, the lack of activity may originate from structural alterations within the catalytic cleft or within the phosphoryl group binding loop when compared to active orthologous or homologous PPIases [38,39]. Goh et al. mentioned that the conservation of residues in the β2-α4 region of *Tb*Par42, which is part of the substrate-binding pocket, is low when compared to homologous regions of other parvulins. According to our structural model of *Tb*Par42-PPIase, there are two significant alterations of residues in the catalytic core region of the protein when compared to *h*Pin1. Position 350 of the proline binding pocket is taken by a tyrosine rather than a methionine, and position 374 of the catalytic site is captured by a leucine (serine in *h*Pin1). To examine the role of these residues on the activity of the PPIase, we mutated these positions in *Tb*Par42 (*Tb*Par42-PPIase_Tyr350Met_, *Tb*Par42-PPIase_Leu374Ser_) and *h*Pin1 (*h*Pin1_Met130Tyr_, *h*Pin1_Ser154Leu_), and tested the catalytic efficacy of the mutants in a protein coupled assay (Figure 5). As *h*Pin1_Met130Tyr_ was still active (Figure 5C), this mutation is not expected to be responsible for the lack of activity in *Tb*Par42-PPIase. This was in accordance with a still inactive *Tb*Par42-PPIase_Tyr350Met_ mutant (Figure 5A). As expected [35], the mutant *h*Pin1_Ser154Leu_ showed no catalytic activity (Figure 5D). However, no gain-of-function for the *Tb*Par42-PPIase_Leu374Ser_ mutant was observed. 

Beside residues in the catalytic cleft and proline binding pocket, three basic amino acids (basic triad) residing in the β1-α1-loop in Pin-type parvulins are functionally important in *h*Pin1. By binding to the phosphoryl group of the substrate, these residues serve as anchor points for the bond rotation occurring in the isomerization step (reviewed in Reference [9]). Thus, not only absence of this basic triad, but changes in their arrangement and its relation to the spatial organization of residues in the catalytic center or in the proline-binding pocket may abolish catalytic activity. In *Tb*Par42, the triad of these putative phosphoryl-binding residues Lys275, Arg280 and Arg281 is conserved with respect to sequence position. Despite this fact, and in contrast to *Tb*Pin1 [40] and other Pin1-typ parvulins, no significant chemical shift perturbations were detected in a HSQC spectrum for resonances of the basic triad of the β1-α1 phosphoryl group binding loop of *Tb*Par42-PPIase (Figure 4) upon addition of a phosphorylated peptide. This indicates the absence of an interaction of the triad with the phosphate group of the peptide. We wondered if structural differences in the arrangement of this loop with relation to the catalytic active site residues would prevent binding of the phosphoryl group. Goh and coworkers suggested that an insertion in the β1-α1 region of the protein may affect catalytic activity. To uncover discrepancies in the spatial arrangement, we compared the overall fold of *Tb*Par42-PPIase to those of the known phosphate-binding relatives *Tb*Pin and *h*Pin1. 

**Figure 6:**
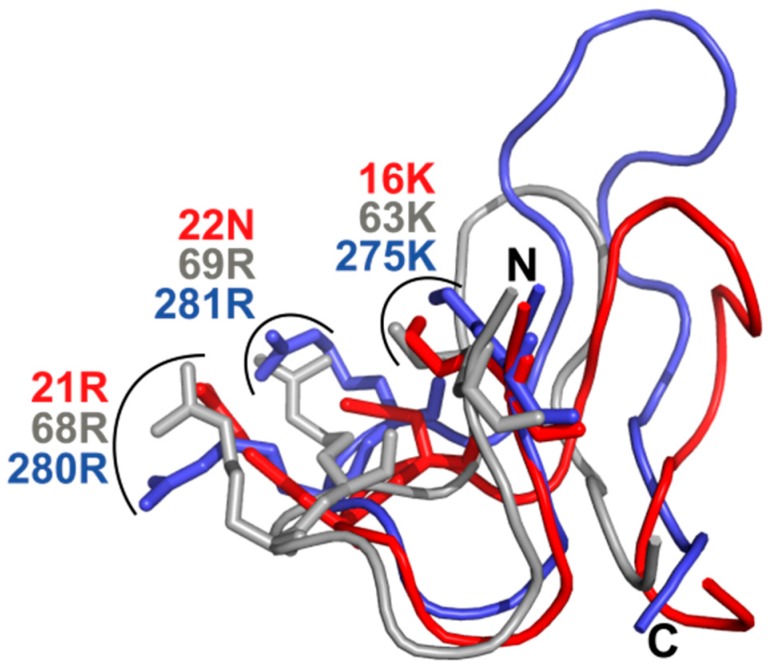
Spatial orientation of the β1-α1 loop regions when superimposing the catalytic core residues of *Tb*Par42 (H271, C333, L374, H377), *Tb*Pin1 (H11, C68, S109, H112) and *h*Pin1 (H59, C113, S154, H157); *Tb*Par42 (PDB:6GMP) (blue), *h*Pin1 (PDB:1NMW) (gray), and *Tb*Pin1 (PDB:2LJ4) (red).

As we wanted to focus on the structural orientation of the side chains of the basic triad and only few NMR constraints (NOEs) exist for the side chain atoms of these residues, we complemented our structural data by crystalizing the *Tb*Par42-PPIase and resolving its structure using X-ray analysis (PDB-code: 6GMP; Table S2) (see also Figures S4 and S5). After superimposition of the catalytic core residues (Figure 6), the spatial orientation of the proteins does not differ with respect to position and orientation. This excludes that the arrangement of phosphoryl group binding residues is responsible for the absence of activity within *Tb*Par42. Thus, the structural reason for the absence of activity remains unknown. To our knowledge, no parvulins from higher eukaryotic organisms have been discovered so far, of which the PPIase domains lack catalytic activity. However, the bacterial PPIase domains of the periplasmic chaperones PpID [41], as well as one PPIase domain of SurA (Par1) [42], are also lacking catalytic activity. Similar to the PPIase domain of *Tb*Par42, the isomerase activity of PpID “could not be generated by substitutions at the peptide binding site” [41]. In case of SurA Par1, the domain was found to cooperate with the second domain Par2 in substrate folding reactions.

### 3.7. NMR Structure Calculation of the N-Terminal Extended FHA Domain

**Figure 7:**
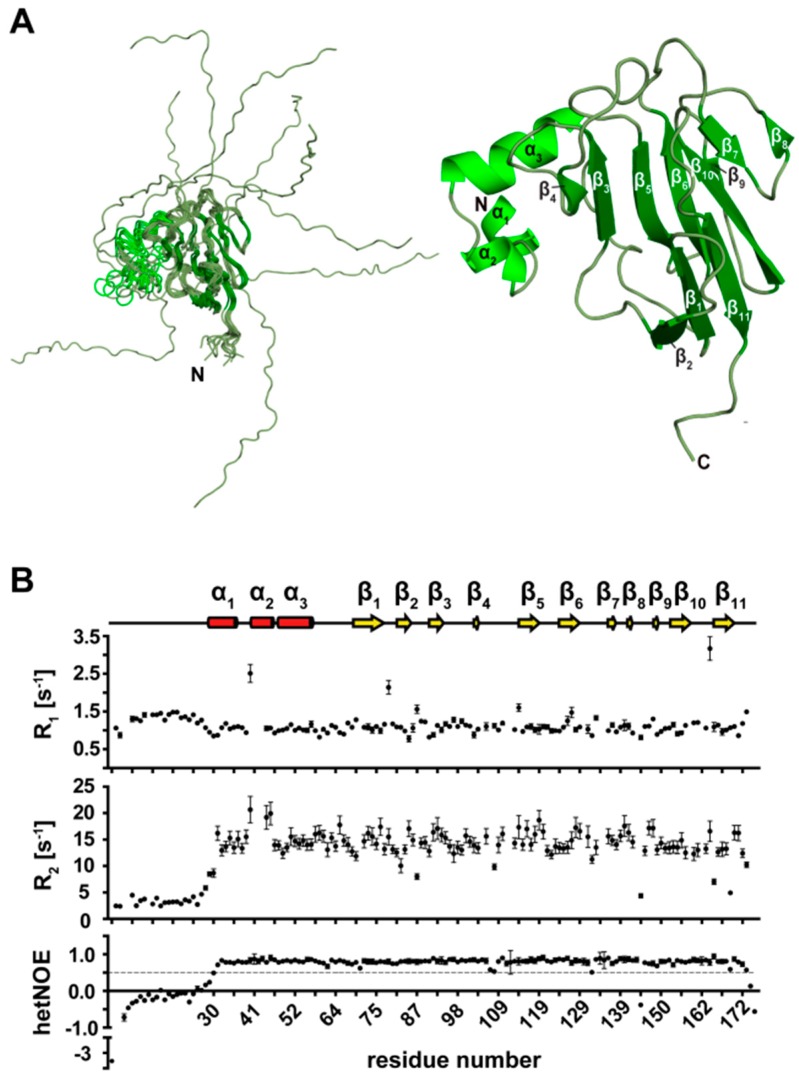
Structural and dynamic data of the FHA domain of *Tb*Par42. (**A**) Ensemble of the 10 lowest energy structures of the N-terminal extended FHA domain (1–177) (left) and ribbon representation of *Tb*Par42-FHA domain without flexible terminal residues 1–33 (right). (**B**) Dynamics of the NterFHA construct. The secondary structure elements are indicated at the top of the plot. Below, the longitudinal (R1) and transversal (R2) relaxation rates, as well as the HetNOE of the amino group of residues within the domain, are plotted.

The solution structure of the NterFHA–domain of *Tb*Par42 was calculated using 2667 distance NOEs, 262 torsion angle restraints, and 42 hydrogen bonds (Table S3). An overlay of the 10 final lowest energy structures is shown in Figure 7A. The ensemble is deposited in the Protein Data Bank (PDB code: 2N84), while chemical assignments and shifts were transferred to the BMRB database with the accession number 25834. Although all 10 structures of the N-terminal extended FHA domain (1–177) fit the experimental restraints, the r.m.s.d value for the backbone atoms of the ensemble was 12.5 Å. It dropped down to 0.33 Å when the flexible terminal residues (1–33) (Figure 7B) and the adjacent three helices α1 (Glu33 to Asn37), α2 (Ile42 to Val45), and α3 (Pro84-Tyr57) were excluded from calculation, indicating an excellent structural resolution of the FHA-domain (residues 58–173). Including α3 in the calculations increased the r.m.s.d value to 0.8 Å for the backbone atoms of the ensemble, while the r.m.s.d value increased to 1.62 Å when additional α1 and α2 were included (Table S3). 

### 3.8. Secondary Structure Elements of the N-Terminal Extended FHA Domain (1–177)

The FHA domain of *Tb*Par42 was analyzed together with the N-terminus of the protein. The low number of long-range NOEs per residue, as well as low R2 amide relaxation values and negative hetNOE data of the first 30 residues within the N-terminus, point towards a random coil conformation of this segment (Figure 7B). Based on distances and typical NOE connectivities, three well-characterized helices α1 to α3 (Glu33–Asn37, Ile42–Val45, Pro48–Tyr57) adjacent to the unstructured region were detected. Via a long loop (Phe58–Ala70), helix α3 is connected to an array of 11 sequentially arranged β-strands. The first β1 strand extends from Cys71 to Arg77. The two following strands, β1 and β2, are both four residues long (Leu82 to Gly86 and Phe92–Gly96), whereas β4 covers just two residues (Tyr103 to Val104). β5 starts at Ala115 and extends to His120. β6 (Cys125–Asp130) is connected to β5 via a turn (Gly121–Arg124). The chemical shift index indicates a helix between strands β6 and β7 (Val137–Leu139), but, due to the fast exchanging amide protons and the missing helical NOE connections, the stretch (Leu131–Gly136) corresponds rather to a turn region. Hydrogen bonds to the other strands helped to identify β-strands β8 (Asn142–Arg143) and β9 (Pro149–Leu15). The last two strands extend from Gly155 to Phe160 (β10) and from Val166 to Leu171 (β11). Residues (Gly172–Ser177) following the last β-strand exhibit random coil character.

### 3.9. Tertiary Structure of TbPar42-NterFHA_1–177_

Like other known FHA structures, the FHA of *Tb*Par42 adopts the typical β-sandwich fold, which is formed by two twisted β-sheets consisting of five (β2-β1-β11-β10-β9) and six (β4-β3-β5-β6-β7-β8) β-strands, respectively, Figure 3B. In both β-sheets, the adjoining β-strands are oriented antiparallel to each other, with the exception of β4, which proceeds parallel to the neighboring β3. Due to ^15^N relaxation data, the loops connecting β-strands 4/5 and 6/7 exhibit a higher flexibility compared to the other loops or turns within the FHA structure. Except for the 30 N-terminal residues and a few residues at the very C-terminal end, the structure has an overall rigid character. In contrast to the compact β-stranded fold, there are only few long-range NOE restraints emerging from the helical part of the protein (Glu33 to Tyr57). Therein, Gln54 of α3 exhibits more than 40 NOE connectivities (e.g., to Phe93 of β3 and to residues Val100, Cys101 and Asp102 within the β3-β4 loop). According to its molecular interactions, α3 is tightly attached to the β-stranded FHA core, in contrast to α1 and α2, where such NOE contacts are absent. 

### 3.10. TbPar42-NterFHA_1–177_ binds pThr-Pro Moieties within Peptides

**Figure 8:**
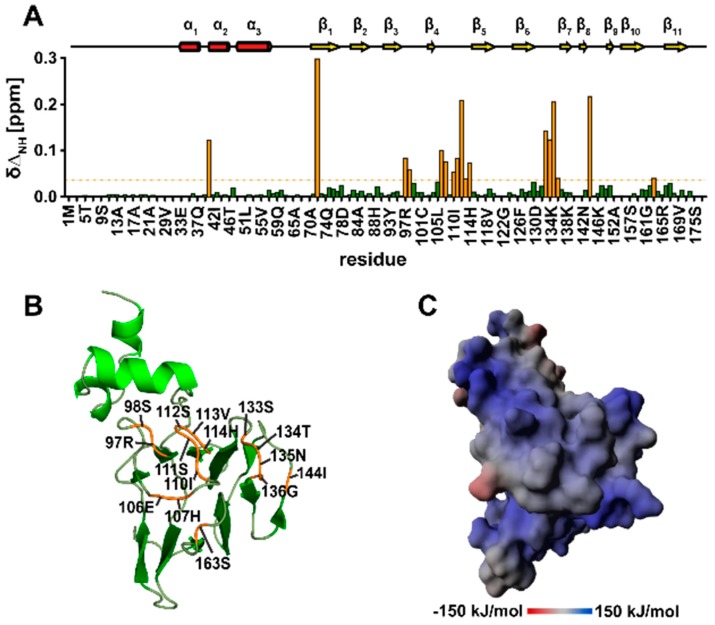
The phosphorylated peptide Suc-Ala-pThr-Pro-Ala-NH_2_ is recognized by conserved phosphate binding loops of *Tb*Par42-FHA. (**A**) (top) Secondary structure elements of NterFHA. (bottom) Diagram showing NH chemical shift changes (∆δ_NH_) versus the amino acid sequence of *Tb*Par42_NterFHA_1–177_ domain after addition of 13 mM peptide. Chemical shift perturbations observed for His41 and His72 may reflect tiny changes in pKs values, rather than indicating strong effects upon peptide addition [35]. Residues with a chemical shift larger than 0.04 ppm (orange dash line) are colored orange. (**B**) Residues of *Tb*Par42-FHA_33–177_ showing chemical shift changes larger than 0.04 ppm in the presence of phosphorylated peptide are indicated and are orange colored. (**C**) Electrostatic surface of the NterFHA_33–177_ generated with YASARA using the Particle Mesh Ewald method. The intensity of the electrostatic potential is gradually colored from dark red (negative) over grey (neutral) to dark blue (positive), representing energy levels from −150 to +150 kJ/mol. The orientation of the structure is as in (**B**).

As FHA domains are known as phospho-threonine recognition and binding modules [5], we examined the affinity of the ^15^N labeled NterFHA to the phosphorylated model peptide Suc-Ala-pThr-Pro-Ala-NH_2_ by performing NMR titration experiments. Within the recorded 2D ^1^H-^15^N HSQC spectrum, the presence of peptide affected the chemical shifts of resonances from residues of several loop-regions of the FHA core, Figure 8A. Perturbed chemical shifts were observed for residues in the β6-β7 loop (Ser133–Gly136), for isoleucine 144 in the β8-β9 loop, serine 112 and surrounding residues in the β4-β5 loop (Glu106, His107, Ile110–His114), for arginine 97 and serine 98 in the β3-β4 loop, and for serine 163 in the β10-β11 loop, Figure 3D. Structural and sequential alignments with other FHA domains indicate that perturbation mainly affects those resonances which are either essential for direct binding to phosphopeptide ligands, or for the maintenance of the binding surface (Figure 8B). Arginine 97 from the β3-β4 loop as well as serine 112 from the β4-β5 loop are expected to form a hydrogen bond network with the phosphate group of the ligand’s pThr moiety. The conserved glycine 96 and histidine 114 at the end of β3 and adjacent to strand 5 are most likely crucial for the structural stability of the binding site [43]. A significant chemical shift perturbation could also be observed for Asn135 and surrounding residues in the β6-β7 loop. In some FHA domains, like in Kanadaptin [44], this position is substituted by a histidine residue. The asparagine/histidine is important for interactions to residues flanking the pThr in the ligand [45]. Although the *Tb*Par42-FHA binds the above mentioned phosphorylated model peptide, binding affinity is in the lower millimolar range (~4 mM). 

Tight binding of FHA domains to phosphorylated motifs greatly depends on the ligand’s residues flanking the phosphorylated threonine, particularly on the third position (pT+3) following pThr [43]. According to this so called “pT+3” rule, FHA domains can be divided in pTxxD or pTxxI/V (x representing any amino acid) binding modules. To test if *Tb*Par42-FHA has a preference for a certain residue at this position, we generated synthetic peptides and tested their binding affinity towards the FHA domain by performing NMR titration experiments. As the proposed peptide-binding surface of the FHA is positively charged (Figure 8C, we used the sequence Ala-Glu-Ala-pThr-Glu-Ala-Xaa as the peptide scaffold and generated derivatives by altering position Xaa (Asp, Ile, Val, Ser, or Gln). Addition of these peptides to the protein perturbs NMR resonances of residues located in the same regions as observed on binding of Suc-Ala-pThr-Pro-Ala-NH_2_, but, in addition, induces chemical shift changes within resonances of amino acids in the β10-β11 loop region. However, no significant improvement in binding affinity compared to the Suc-Ala-pThr-Pro-Ala-NH_2_ peptide could be measured (Table S4). 

### 3.11. The Isolated Linker Region between the NterFHA and PPIase Domain Is Flexible and Unfolded

**Figure 9:**
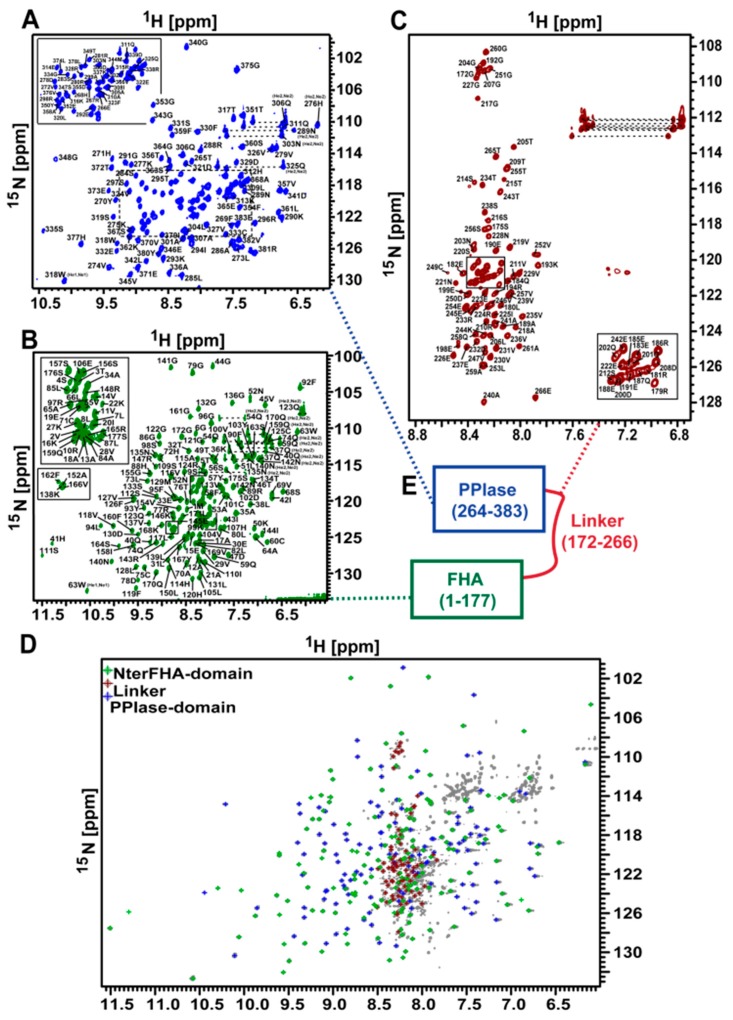
The domains of *Tb*Par42 do not interact with each other (**A**) Fully assigned ^1^H-^15^N-HSQC spectrum of the PPIase domain_264-383_. (**B**) Fully assigned ^1^H-^15^N-HSQC spectrum of the N-terminal extended FHA domain_1–177_. (**C**) 96.6% assigned ^1^H-^15^N-HSQC spectrum of the linker_172–266_ region. (**D**) Overlay of the *Tb*Par42 full-length TROSY spectrum with the HSQC spectra of the separately analyzed domains and the linker of *Tb*Par42. (**E**) Scheme of domain organization.

As we were able to completely assign the amide resonances in the ^1^H-^15^N-HSQC spectra of the NterFHA_1–177_ (Figure 9B) and PPIase_264-383_ (Figure 9A) domains, we expressed and purified the ^13^C-^15^N-labeled linker region (residues 172–266) connecting the two domains. The signal dispersion in the ^1^H-^15^N-HSQC spectrum, as well as the chemical shift index analysis of the linker, indicate that the region is mostly random coil (Figure 9C). The backbone amide groups of the N-terminal stretch Pro174–Lys178 of the linker are absent in the ^1^H-^15^N-HSQC spectrum, resulting in 96.6% assignment completeness. NMR shift data indicate a long α-helix extending from Arg179 to Val197 (Figure S1). Upon calculation of a Rosetta model (Figure S2) [46], five putative transient helices are predicted within this linker regions.

### 3.12. There Is No Interaction between the Domains of TbPar42

Due to the high molecular weight of the protein, we analyzed the domains of *Tb*Par42 as isolated constructs. However, the structures and activities of these isolated domains may be modulated by intra-domain interactions within the protein. To investigate such putative modulations, the 2D-TROSY spectrum of triple labeled ^2^H-^13^C-^15^N-*Tb*Par42 protein was superimposed with the assigned ^1^H-^15^N-HSQC spectra of the isolated constructs recorded under identical conditions, Figure 9D. Interaction of domains should lead to significant chemical shift changes in the full-length protein when compared to its isolated domains. However, beside a few terminal residues of the isolated constructs, 353 signals from all three HSQC spectra perfectly matched to the TROSY spectrum, indicating that there are no interactions of the individual domains nor conformational rearrangements within the linker region under the tested buffer conditions.

## 4. Discussion

### 4.1. What is the Cellular Role of the FHA Domain of TbPar42?

*Tb*Par42_1–177_ resembles the structural fold of a typical FHA domain. Multi-domain proteins where a FHA domain precedes a parvulin PPIase domain are unique to unicellular organisms (e.g., *Trypanosoma* and *Leishmania* [2], as well as in *Chlamydomonas* [3] and *Dyctiostelium* [2]). The FHA domain of *Tb*Par42 seems to be involved in the binding of phosphorylated target proteins, as could be proven by NMR titration experiments with model ligand peptides. The dissociation constants of the tested peptides, however, were only in the millimolar range, demonstrating most likely that the optimal binding sequence for this protein domain remains to be elucidated. Nevertheless, binding occurred in regions found to interact with target proteins in homologous and related FHA structures (Figure 8A,B). As the *Tb*Par42 protein is unique to parasitic protozoa organisms and no interaction partners have been published so far, we can only speculate about its functional role by relying on similar and orthologous domains and proteins in other organisms.

In order to find orthologous proteins for the FHA domain of *Tb*Par42, we ran a DALI-search that depicted the highest Z-score (13 and 12.2), as well as the highest sequence identity (37%), to the FHA domains of the proteins Kanadaptin (human) and DAWDLE (DDL; *Arabidopsis thaliana*). Both representatives are nuclear-localized proteins. While almost nothing is known about the function of Kanadaptin, DDL appears to act in multiple developmental processes such as growth of root and shoot, as well as in floral morphogenesis and fertility [47]. Dawdle was reported to stabilize the interaction between the protein Dicer-like 1 (DCL1) and a pre-miRNA [48], and therefore is involved in the biogenesis of small RNAs and in gene silencing. Its human orthologue, Smad nuclear interacting protein 1 (SNIP1), has been attributed a similar function [48]. Interestingly, the human *h*Par14 parvulin protein was also found to be involved in ribosome biogenesis and RNA processing [49], and binds to double-stranded nuclear acids too. In addition to the cellular functions of DDL in Arabidopsis, the human SNIP1 constitutes an inhibitor of TGF-β and NF-κB signaling pathways by competing with the TGF-β canonical signaling protein Smad4 and the NF-κB transcription factor p65/RelA for binding to the transcriptional coactivator p300 [50,51]. Noticeably, human parvulins also act in these signaling pathways. *h*Pin1 protein has been demonstrated to be involved in a variety of these signaling events, e.g., of the canonical (Smad signaling) and non-canonical (Ras/ERK and PI3K/Akt) pathways of TGF-β (as reviewed in References [52,53]) and of NFκB [54]. In addition, *h*Par14 was found to act in insulin activated PI3K-Akt signaling (reviewed in Reference [9]). The fact that human parvulins are involved in cellular events in which orthologous proteins of the parvulin *Tb*Par42 are also involved may lead towards a role of *Tb*Par42 in TGF-β and NFκB signaling, as well as RNA biogenesis and stability. These activities may be important for *Trypanosoma* to control host defense mechanisms and immunity.

### 4.2. TbPar42 May Interact as a Protein Recruitment Platform

**Figure 10:**
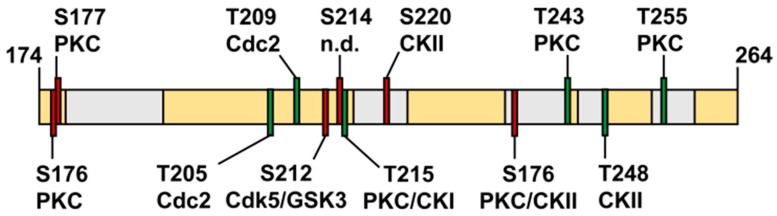
Phosphorylation sites predicted by Netphos-3b1. Schematic drawing of the linker region (residues 174 to 264) in yellow. Putative helices are represented by gray horizontal bars. Predicted phosphorylation sites are depicted as red (serine) and green (threonine) vertical bars. The respective phosphorylated residues and their putative modifying kinases are marked (PKC = protein kinase C; CKI/CKII = casein kinase I/II; Cdc/Cdk = cyclin-dependent kinase; GSK3 = Glycogensynthase kinase 3).

The domains of *Tb*Par42 are lined up as a string of pearls. They seem to act independently of each other, or they may interact only after binding to a yet unknown binding partner. Following this hypothesis, *Tb*Par42 would function as a specific dynamic recruitment platform, a scaffold protein that allows weakly associating proteins to be engaged in higher order assemblies [55]. Such assemblies allow a cell to gain spatiotemporal control over protein activity. Many cellular events, such as signal transduction cascades or gene activation and its control processes, involve such transient buildup of higher-order protein complexes. SNIP1 and the related eukaryotic parvulin proteins such as *h*Par14 and *h*Par17 have already been demonstrated to be involved in such events and assemblies [49,56,57,58]. An alternative explanation for the absence of domain interaction involves post-translational modification (PTM) of the protein, especially of its linker region. A Netphos-3.1 data search [59] predicted a plethora of phosphorylation sites within this region (Figure 10). In the N-terminus, only one such site (Thr32 by CKII) was found. 

Most of the detected phosphorylation sites in the linker are at the beginning or within the helical regions predicted in our Rosetta model (Figure S2). Post-translational modifications often act in stabilizing transient helices, or in preventing the formation of such secondary structure elements. The amount and sequential arrangement of PTMs within a mainly unstructured large linker region (according to our NMR data) characterize this part of the protein as intrinsically disordered region (IDR) [60,61]. Such regions play a general regulatory role in signaling and controlling pathways [62,63,64,65]. 

Noticeably, all (but cdc2) kinases, which, according to the Netphos prediction, execute the post-translational modifications of the *Tb*Par42 linker region (Figure 10), are involved in Wnt-signaling [66,67], and are active in cytoskeleton organization, mitotic regulation, neuronal patterning, and cell fate decision. Many of these events have been demonstrated to be influenced by parvulins Pin1 and Par14/17 [9,68,69] in human cells. The IDR character of *Tb*Par42 and the involvement of relatives from other organisms in protein assemblies seems to be supportive for the recruitment platform hypothesis of this protein.

## Supplementary Materials

The following are available online at https://www.mdpi.com/2218-273X/9/3/93/s1, Table S1: Structural statistics for the deposited ensemble of 10 lowest energy PPIase structures, Table S2: Structural statistics for the deposited PPIase structure solved by X-ray analysis, Table S3: Structural statistics for the deposited ensemble of 10 lowest energy NterFHA structures, Table S4: Mean K_D_ values after titration of Ala-Glu-Ala-pThr-Ala-Glu-Xaa-peptides and Suc-Ala-pThr-Pro-Ala-NH_2_ to NterFHA of *Tb*Par42, Table S5: Oligonucleotides used as forward and reverse primers for PCR-amplification of the constructs. Figure S1: NMR shift data of the linker region of *Tb*Par42 (A) and secondary structure predictions, Figure S2: CS Rosetta model of the *Tb*Par42 linker region, Figure S3: Sequence alignment of the PPIase domains of *Tb*Par42 and *h*Pin1, Figure S4: NMR structure (PDB-ID: 2N87) of the PPIase domain of *Tb*Par42, Figure S5: Comparison of X-ray structure (PDB-ID: 6GMP) and NMR structure (PDB-ID:2N87) of the PPIase domain of *Tb*Par42.
