# Supplementary material for: Structural Analysis of the 42 kDa Parvulin of Trypanosoma brucei
Source: Biomolecules. 2019 Mar 7;9(3):93. doi: 10.3390/biom9030093 (PMC6468809; doi:10.3390/biom9030093)
Supplement: Supplementary file 1 [file biomolecules-09-00093-s001.pdf]

Structural Analysis of the 42 kDa Parvulin of *Trypanosoma brucei*

Edisa Rehic[1], Dana Hoenig[1], Bianca E. Kamba[1], Anna Goehring[1], Eckhard Hofmann[2], Raphael Gasper[2,3], Anja Matena[1] and Peter Bayer[1]

[1]University Duisburg-Essen, Research Group Structural and Medicinal Biochemistry, Centre for Medical Biotechnology (ZMB), University of Duisburg-Essen, 45117 Essen, Germany

[2]Protein Crystallography, Faculty of Biology and Biotechnology, Ruhr University Bochum, 44801 Bochum, Germany

[3]Max-Planck-Institute for Molecular Physiology, 44227 Dortmund, Germany

Supporting Information

Table S1. Structural statistics for the deposited ensemble of 10 lowest energy PPIase structures (PDB-ID:2N87).

NOE distance constraints	3023
Intraresidual und sequential $\|i-j\|<=1$	1910
Medium range, $1<\|i-j\|<5$	408
Long range $\|i-j\|>=5$	705
Torsion angle constraints	206
Φ-angle	103
Ψ-angle	103
Hydrogen bonds	46
Ramachandran plot (PPIase264-383)	
Residues in most favored regions (%)	78.8
Residues in allowed regions (%)	21.2
Residues in disallowed regions (%)	0
Root mean square deviation (RMSD [Å]) to the mean structure	
Backbone atoms	0.41 ± 0.08 Å
Heavy atoms	0.93 ± 0.05 Å

Table S2. Structural statistics for the deposited PPIase structure (PDB-ID: 6GMP) solved by X-ray analysis. (Numbers in parenthesis represent highest resolution shell)

Beam line	PETRA III, PX13, DESY, Hamburg, Germany
Cell Parameters [Å]	28.6 69.9 102.9, 90° 90° 90°
Space group	C2221
Wavelength [Å]	0.971
Resolution [Å]	18.46 – 1.35 (1.41-1.35)
No. of reflections	121870 (2064)
Unique reflections	23777 (1076)
Redundancy	5.1
Redundancy value for highest shell	2.4 (at 1.35 Å)
I / σ	36.77 (1.19)
I/σI	1.19 at 1.35 Å
CC1/2 [%]	100 (48.6)
Rmeas [%]	2.3 (83.7)
Wilson B-Factor [Å ^{2}]	22.18
Refinement	
Rwork / Rfree [%]	14.6 / 18.1
r.m.s.d. bond length [Å]	0.006
r.m.s.d. angles [°]	1.135
Ramachandran favoured (allowed, outliers) [%]	98.4 (1.6, 0)
Mean B-Factor [Å ^{2}]	32.45
No. of atoms (with H)	
Total	2107
Water	135

Table S3. Structural statistics for the deposited ensemble of 10 lowest energy NterFHA structures (PDB-ID: 2N84)

NOE distance constraints	2667
Intraresidual und sequential |i-j|<=1	1724
Medium range, 1<|i-j|<5	359
Long range |i-j|>=5	584
Torsion angle constraints	262
Φ-angle	131
Ψ-angle	1031
Hydrogen bonds	42
Ramachandran plot (PPIase264-383)	
Residues in most favored regions (%)	80.1
Residues in additional allowed regions (%)	19.9
Residues in generously allowed regions (%)	0.1
Residues in disallowed regions (%)	0
Root mean square deviation (RMSD [Å]) to the mean structure 1-177	
Backbone atoms	12.5 ± 4.2 Å
Heavy atoms	12.8 ± 4.5 Å
Root mean square deviation (RMSD [Å]) to the mean structure 33-177	
Backbone atoms	1.62 ± 0.52 Å
Heavy atoms	1.89 ± 0.51 Å
Root mean square deviation (RMSD [Å]) to the mean structure 48-177	
Backbone atoms	0.80 ± 0.15 Å
Heavy atoms	1.02 ± 0.12 Å
Root mean square deviation (RMSD [Å]) to the mean structure 58-173	
Backbone atoms	0.33 ± 0.07 Å
Heavy atoms	0.74 ± 0.06 Å

Table S4. Mean K_D values calculated from ^1H-^{15}N-HSQC experiments after adding unlabeled Ala-Glu-Ala-pThr-Ala-Glu-Xaa-peptides (up to 8 mM) or Suc-Ala-pThr-Pro-Ala-NH_2 (up to 13 mM) stepwise to a *Tb*Par42 NterFHA solution (250 μM).

	**Ala-Glu-Ala-pThr-Ala-Glu-Xaa**					**Suc-Ala-pThr-Pro-Ala-NH_2**
**Xaa**	**Ser**	**Asp**	**Val**	**Ile**	**Glu**	
**K_D [mM]**	3.1	3.4	3.9	4.1	5.4	5.4

Table S5. Oligonucleotides used as forward and reverse primers for PCR-amplification of the constructs *Tb*Par42-full length (1-383), N-terminal extended FHA domain (NterFHA) (1-177), linker region (172-266) and PPIase domain (264-383) from the synthetized *Tb*Par42 DNA as a template.

**constructs**	**oligonucleotides**
*Tb*Par42-FL	CACACA GGGCCC ATGGTGACAAGC
	GGTTGG CTCGAG CTTA TTCCACGCGA
*Tb*Par42-NterFHA	CACACA GGGCCC ATGGTGACAAGC
	GGTTGG CTCGAG CTTA AG AAG AAG ACG GTG GCC CC
*Tb*Par42-Linker	CACACA GGGCCC GGG CCA CCG TCT TCT TCT AA
	GGTTGG CTCGAG CTTA TTC GGT CGG CGG CGG C
*Tb*Par42-PPIase	GGTTGG CTCGAG CTTA TTCCACGCGA
	CACACA GGGCCC CCG ACC GAA CGC CAT TTT TAT

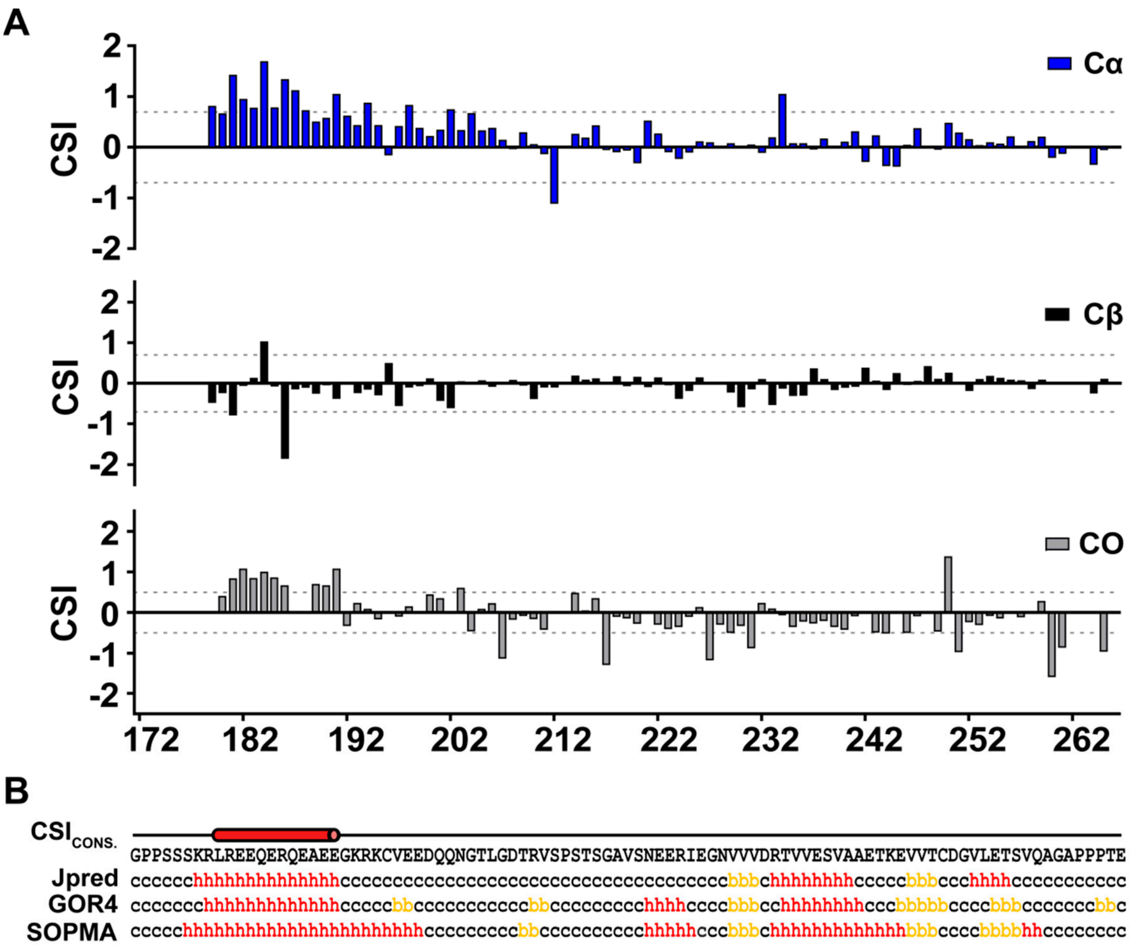

Figure S1. NMR shift data (chemical shift index) of the linker region of *Tb*Par42 (A) and the CSI consensus with the linker sequence from aa 172 to aa 266 and the secondary structure predictions by Jpred4 [1] , GOR4 [2] and SOPMA [3] (B)

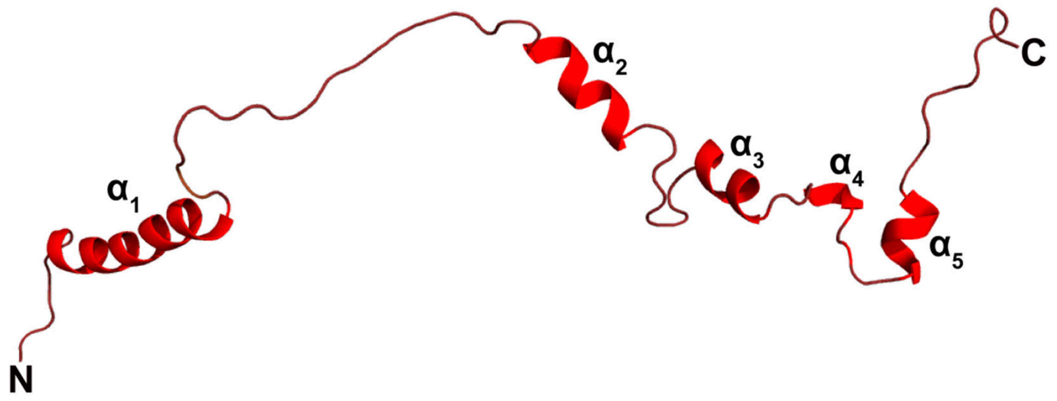

Figure S2. CS Rosetta model of the *Tb*Par42 linker region (Arg179-Glu266) calculated on the server of the BMRB data bank (https://csrosetta.bmrb.wisc.edu/). A representative structure (number 10 from the top ten ensemble) out of 3000 calculated models is shown. The helices presented in this figure are conserved (minor alterations in length) within all models of the top ten ensemble. Calculation was performed on basis of H_N, Cα, Cβ, CO and N chemical shifts extracted from NMR spectra. During calculation, flexible regions were not removed within the flexible tail handling procedure, but were disregarded during energy minimization. Owing to the flexibility of the linker region no r.m.s.d values are provided.

```
TbPar42          264 PTERHFYHVLVKHKDVRRPSSLAPRNKGEKITRSRADAINLAQAILAQHK    313
                     |......|:||||...|||||.    :.|||||::.:|:.|....:.:.|
hPin1             52 PARVRCSHLLVKHSQSRRPSSW----RQEKITRTKEEALELINGYIQKIK     97

TbPar42          314 ERKTWSLDEFVQVVRDFSECGSAKRDGDLGMVESGTYTEGFDTVAFSLKS    363
                     ..:    ::|..:...||:|.|||..||||....|...:.|:..:|:|::
hPin1             98 SGE----EDFESLASQFSDCSSAKARGDLGAFSRGQMQKPFEDASFALRT    143

TbPar42          364 GEVSAPVETELGVHLIYRVE    383
                     ||:|.||.|:.|:|:|.|.|
hPin1            144 GEMSGPVFTDSGIHIILRTE    163
```

Figure S3. Sequence alignment of the PPIase domains of *Tb*Par42 (blue) and *h*Pin1 (grey). The pairwise sequence alignment was produced using the selected EMBOSS tool Needle (https://www.ebi.ac.uk/Tools/psa/emboss_needle/) [4]. We used the matrix EBLOSUM62, a gap penalty of 10.0 and an extended penalty of 0.5. A sequence identity of 37.5% (bold letters, symbol = lines) and a sequence homology of 56.7% (symbols = dots and colons) with 6.7% of gaps were calculated by the program (Score = 210).

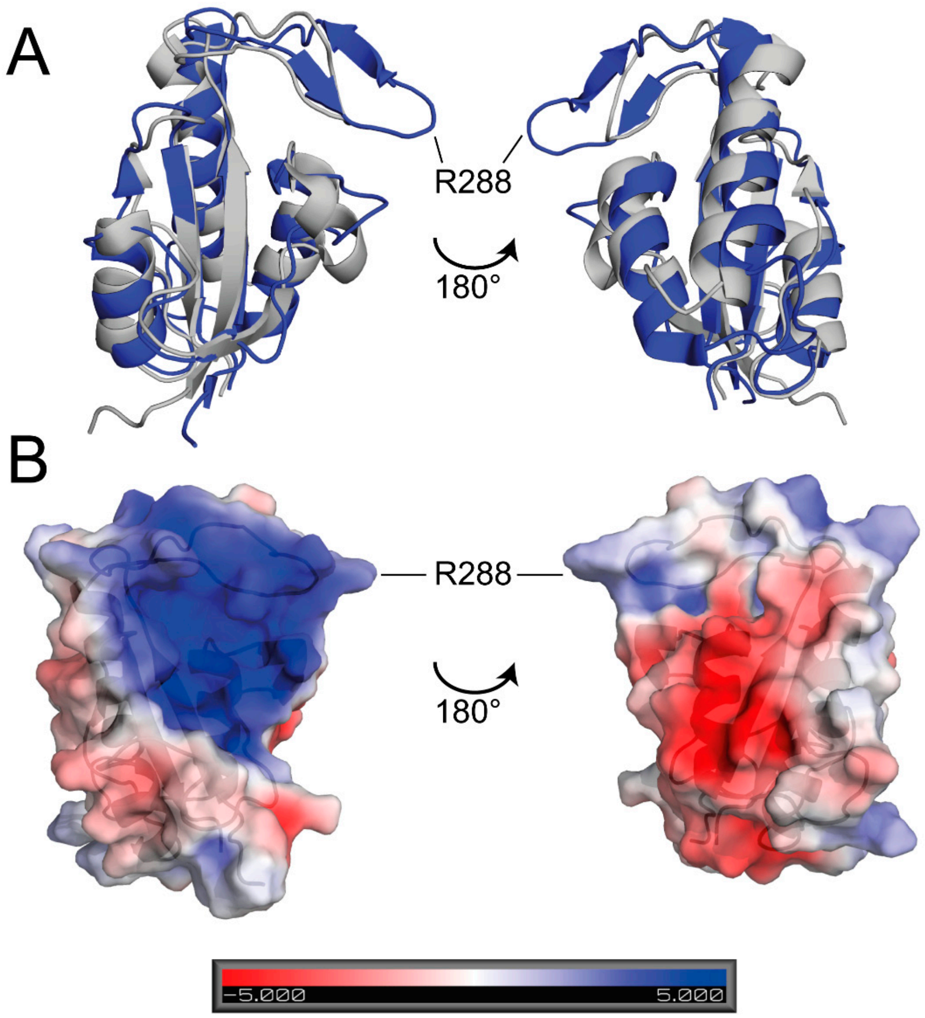

Figure S4. NMR structure (PDB-ID: 2N87) of the PPIase domain of *Tb*Par42. A, Superposition of the PPIase domains of *Tb*Par42 (blue) and *h*Pin1 (grey) (PDB-ID:1NMW). The position of the outmost α1-β2 loop residue Arg288 is labeled. Structures were superimposed using the PyMOL Molecular Graphics System, Version 1.3. B, Surface electrostatics of *Tb*Par42 PPIase domain. Electrostatics calculated by APBS (the Adaptive Poisson-Boltzmann Solver) in PyMol. The partial charges and atom radii for the calculation was generated by PDB2PQR Version 2.0.0. The electrostatic surface is color-coded (from -5 k_BT/e_c to +5 k_BT/e_c)

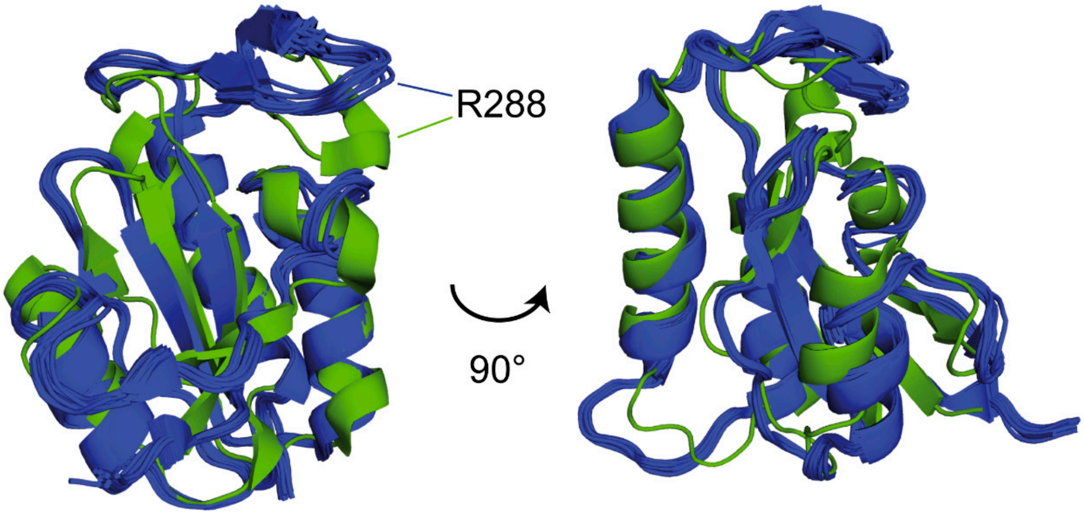

Figure S5. Comparison of X-ray structure (PDB-ID: 6GMP) and NMR structure (PDB-ID:2N87) of the PPIase domain of *Tb*Par42. The X-ray structure (green) was superimposed with the NMR ensemble (blue) using the program PyMol. The overall Cα r.m.s.d. of the alignment is 2.43 Å, the r.m.s.d. over all heavy atoms is 3.75 Å. The position of the outmost α1-β2 loop residue Arg288 is labeled.

References

1. Drozdetskiy, A.; Cole, C.; Procter, J.; Barton, G.J. JPred4: a protein secondary structure prediction server. *Nucleic Acids Research* **2015**, *43*, W389-94, doi:10.1093/nar/gkv332.

2. Garnier, J.; Gibrat, J.-F.; Robson, B. [32] GOR method for predicting protein secondary structure from amino acid sequence. In *Computer methods for macromolecular sequence analysis*; Doolittle, R.F., Ed.; Acad. Press: San Diego, Calif., 1996; pp 540–553, ISBN 9780121821678.

3. Geourjon, C.; Deléage, G. SOPMA: significant improvements in protein secondary structure prediction by consensus prediction from multiple alignments. *Computer applications in the biosciences : CABIOS* **1995**, *11*, 681–684.

4. Rice, P.; Longden, I.; Bleasby, A. EMBOSS: The European Molecular Biology Open Software Suite. *Trends in Genetics* **2000**, *16*, 276–277, doi:10.1016/S0168-9525(00)02024-2.
